# Biochemical and phenotypic characterisation of the *Mycobacterium smegmatis* transporter UspABC

**DOI:** 10.1016/j.tcsw.2021.100052

**Published:** 2021-04-24

**Authors:** Magdalena Karlikowska, Albel Singh, Apoorva Bhatt, Sascha Ott, Andrew R. Bottrill, Gurdyal S. Besra, Elizabeth Fullam

**Affiliations:** a School of Life Sciences, University of Warwick, Coventry CV4 7AL, UK; b Institute of Microbiology & Infection, School of Biosciences, University of Birmingham, Birmingham B15 2TT, UK; c Warwick Medical School, University of Warwick, CV4 7AL, UK; d Bioinformatics Research Technology Platform, University of Warwick, Coventry CV4 7AL, UK

**Keywords:** Mycobacteria, ABC-transporter, UspABC, Carbohydrates

## Abstract

*Mycobacterium tuberculosis* (*Mtb*) is an intracellular human pathogen that has evolved to survive in a nutrient limited environment within the host for decades. Accordingly, *Mtb* has developed strategies to acquire scarce nutrients and the mycobacterial transporter systems provide an important route for the import of key energy sources. However, the physiological role of the *Mtb* transporters and their substrate preference(s) are poorly characterised. Previous studies have established that the *Mtb* UspC solute-binding domain recognises amino- and phosphorylated-sugars, indicating that the mycobacterial UspABC transporter plays a key role in the import of peptidoglycan precursors. Herein, we have used a wide array of approaches to investigate the role of UspABC in *Mycobacterium smegmatis* by analysis of mutant strains that either lack the solute binding domain: Δ*uspC* or the entire transport complex: Δ*uspABC*. Analysis of mycobacterial transcripts shows that the *uspABC* system is functionally expressed in mycobacteria as a contiguous reading frame. Topology mapping confirms an N_in_-C_in_ orientation of the UspAB integral membrane spanning domains. Phenotypic microarray profiling of commercially available sugars suggests, unexpectedly, that the *uspC* and Δ*uspABC* mutants had different carbon utilisation profiles and that neither strain utilised glucose-1-phosphate. Furthermore, proteomics analysis showed an alteration in the abundance of proteins involved in sugar and lipid metabolism, crucial for cell envelope synthesis, and we propose that UspABC has an important role in determining the interplay between these pathways.

## Introduction

*Mycobacterium tuberculosis* (*Mtb*) is a major human pathogen and is the causative agent of tuberculosis (TB). *Mtb* is an extremely successful pathogen and TB is one of the leading causes of death world-wide from a bacterial pathogen (World Health Organisation, 2020). In 2019 alone, approximately 1.5 million people died from TB and over 10 million new TB cases were reported (World Health Organisation, 2020). TB is treatable and curable, however effective treatment regimens have been threatened and rendered ineffective by the emergence of drug-resistant strains of *Mtb*. Therefore, there is an urgent need to identify novel targets and pathways within *Mtb* to develop new therapeutic interventions to address this global health problem.

*Mtb* is an intracellular pathogen with a remarkable ability to survive for decades in a nutrient restricted environment within the human host. This feature is attributed, in part, to the highly complex mycobacterial cell envelope that distinguishes *Mtb* from other prokaryotic species (Brennan, 2003, Brennan and Nikaido, 1995). The cell wall core consists of a cross-linked peptidoglycan network covalently attached to an arabinogalactan polysaccharide which in turn is covalently attached to long chain mycolic acids, with additional lipids interspersed within the mycolic acid layer, forming an outer ‘mycomembrane’ (Brennan, 2003, Brennan and Nikaido, 1995). This ‘waxy’ *Mtb* cell envelope poses a significant permeability barrier that prevents many molecules from entering the cytoplasm and is a major factor that contributes to the intrinsic resistance of mycobacteria to most antibiotics (Brennan, 2003, Brennan and Nikaido, 1995, Batt et al., 2020, Abrahams and Besra, 2021). Furthermore, the mycobacterial cell wall hinders access of critical nutrients and we still lack a complete understanding about the precise energy sources that are available during the *Mtb* infection life-cycle and the systems linked with their import. Previous bioinformatics studies that interrogated the *Mtb* genome have provided information about potential *Mtb* ATP-binding cassette (ABC) transporters involved in the import of nutrients across the inner membrane (Braibant et al., 2000, Niederweis, 2008, Cole et al., 1998). These studies revealed that *Mtb* has 16 putative ABC transporters predicted to be involved in the import of ions, amino acids and carbohydrates (Braibant et al., 2000, Niederweis, 2008). It is particularly notable that the *Mtb* genome encodes for only four ABC-carbohydrate importers, fewer than other bacterial pathogens, and may reflect the availability of a discrete set of sugars within the host environment.

Recently our understanding of the role of the *Mtb* carbohydrate importers has improved and substrates for some of these systems have been identified (Fenn et al., 2019, Fullam et al., 2016, Kalscheuer et al., 2010b, Parker et al., 2020, Furze et al., 2021, Lowery et al., 2015). Interestingly, it has been shown that the *Mtb* LpqY-SugABC transporter specifically recycles trehalose from the mycobacterial cell envelope and is essential for *Mtb* virulence (Kalscheuer et al., 2010b, Furze et al., 2021). Similarly, the *Mtb* UgpB substrate-binding protein belonging to the *Mtb* UgpABCE transporter appears to have an important role in *Mtb* pathogenesis and is up-regulated during infection and recognises a broad selection of glycerophospholipids (Kruh et al., 2010, Somashekar et al., 2011). Structural analysis has revealed the molecular mechanisms involved in glycerophosphodiester recognition, which are likely to be derived from the lipolysis of membrane phospholipids (Fenn et al., 2019). The functional role and substrate specificity of the Rv2038c-2041c transporter is not yet known, however the Rv2041c substrate-binding protein is up-regulated under *in vitro* culture conditions that mimic macrophage conditions and the recombinant Rv2041c protein elicits an immune response in mice suggesting that this putative *Mtb* carbohydrate ABC-transporter has a role in host-pathogen interactions (Kim et al., 2009, Kim et al., 2008, Shin et al., 2009).

The physiological role of the *Mtb* UspABC transporter is less clear. However, studies of the *Mtb* UspC substrate domain shows that its overall structural architecture is typical of ABC-transporter substrate binding proteins (Fullam et al., 2016). Biochemical analyses have revealed that *Mtb* UspC has a distinct preference for amino sugars, including D-glucosamine-6-phosphate and chitobiose, a β-1,4 linked glucosamine disaccharide, suggesting a potential role for this transporter in the uptake of recycled sugar components of the mycobacterial cell wall (Fullam et al., 2016). However, the physiological role of the UspABC transporter is not yet known and is the focus of this study. The *Mtb* UspABC transporter (annotated as UspAEC in *Mycobacterium smegmatis:* high sequence identity to the *Mtb* UspABC transporter at the amino acid level (UspA: 83%; UspB: 79%; UspC: 69%) suggesting an overlapping function) is encoded by three genes (Supplementary Fig. S1), of which the first two genes *uspA* and *uspB* encode for the membrane spanning domains of the transporter, while the final gene, *uspC*, encodes for the substrate binding protein (Kapopoulou et al., 2011). There is no obvious genetic association of *uspABC* with a nucleotide binding domain (NBD) to complete the ABC-transporter, however it is possible that the UspABC transporter shares a NBD with other transporters located elsewhere within the genome, which is reported for other bacterial ABC-transporters (Tomii and Kanehisa, 1998, Eitinger et al., 2011, Vahedi-Faridi et al., 2010). Notably, the UspABC transporter is highly conserved across mycobacterial species, including *M. leprae*, which has undergone significant gene decay (Supplementary Fig. S1), resulting in a core set of genes that are considered to be essential for infection (Cole et al., 2001).

The aim of this study was to investigate the physiological role of the UspABC transporter in mycobacteria. To achieve this aim we generated a deletion mutant of *uspC* that encodes the substrate binding protein, and of the entire transporter complex: *uspAEC* and undertook a combination of approaches involving topology mapping, antimicrobial susceptibility testing, phenotypic microarray profiling and proteomics analysis in *Mycobacterium smegmatis*, a non-pathogenic model organism. The combined results demonstrate that a non-functioning mycobacterial UspAEC transporter results in increased sensitivity to vancomycin, distinct carbon source utilisation profiles and alterations in pathways involved in carbohydrate and lipid metabolism.

## Materials and methods

### Bacterial strains, culture conditions and chemicals

All strains were derived from *M. smegmatis* mc^2^155 and routinely cultured aerobically in either Luria-Bertani (LB) supplemented with 0.05% Tween 80, Tryptic Soy Broth (TSB) supplemented with 0.05% Tween 80, Middlebrook 7H9 broth (Difco) supplemented with 10% Albumin‐Dextrose‐Catalase (ADC), 0.2% glycerol and 0.05% Tween 80 or Sauton’s media or on LB-agar or Middlebrook 7H10 agar (Difco) supplemented with 10% oleic acid‐albumin‐dextrose‐catalase (OADC) and 0.2% glycerol at 37 °C. Hygromycin (50 μg/ml), kanamycin (25 μg/ml) and 2.5% sucrose were added for selection for appropriate strains. For growth on defined carbon sources the following minimal media was used: 64 g/L Na_2_HPO_4_, 15 g/L KH_2_PO_4_, 2.5 g/L NaCl, 5 g/L NH_4_Cl in H_2_O, 2 mM MgSO_4_, and 0.1 mM CaCl_2_ and 0.05% (vol/vol) Tyloxapol. For cloning procedures *Escherichia coli* Top10 cells were grown in LB broth or on LB agar with hygromycin (150 μg/ml), kanamycin (50 μg/ml). All plasmid sequences were verified by DNA sequencing (Eurofins-GATC). All chemicals were purchased from Sigma-Aldrich and all PCR and restriction enzymes were obtained from New England Biolab, unless otherwise stated.

### Generation of the *uspC* gene-deletion mutant

The construct for deletion of *uspC* was generated in the pJG1100 allelic exchange vector (a gift from Dr Neeraj Dhar, EPFL, Switzerland). Two DNA fragments ~ 900 bp upstream and downstream of *uspC* were amplified by PCR from *M. smegmatis* genomic DNA using the primers listed in Table S1. The PCR products were digested with PacI/AvrII (upstream fragment) and AvrII/AscI (downstream fragment) and ligated in-frame with the AvrII site with the pJG1100 vector digested with the same restriction enzymes resulting in the *uspC*_pJG1100 construct. Deletion of *uspC* from *M. smegmatis* was achieved by homologous recombination with the *uspC*_pJG1100 vector. *M. smegmatis* mc^2^155 was transformed with *uspC*_pJG1100 and the first recombination event was selected on 7H10 agar containing hygromycin and kanamycin. Integration of the construct at the correct chromosomal locus was confirmed by PCR with the primer pairs listed in Table S1. Deletion of the *uspC* gene was achieved by plating the correctly integrated clones on 7H10 supplemented with 2.5% sucrose for counterselection of the pJG1100 vector. Sucrose resistant clones were screened by colony PCR (Table S1) and the *M. smegmatis* Δ*uspC* mutant strain was confirmed by whole-genome sequencing of genomic DNA (MicrobesNG).

### Generation of the *uspAEC* gene-deletion mutant

The construct for the deletion of the *uspAEC* operon was generated in the digested p0004s vector (a gift from Professor William R. Jacobs Jr, Albert Einstein College of Medicine, USA). Two DNA fragments corresponding to ~ 1000 bases upstream and downstream of *uspAEC* operon were amplified by PCR from *M. smegmatis* genomic DNA using the primers listed in Table S1. The PCR products were digested with AlwNI and ligated with the *hyg^R^*-*sac*B cassette and *ori*E-cos fragments released from the Van91I-digested p0004S vector. The allelic exchange plasmid: *uspAEC*_p0004s, was verified by DNA sequencing, using the primer pairs HL/OL and HR/OR (Table S1). The resulting knockout plasmid was linearized with PacI and cloned into the temperature sensitive phasmid phAE159, as described (Bardarov et al., 2002). Allelic exchange in *M. smegmatis* was achieved by specialised transduction using hygromycin for selection. This resulted in the replacement of *uspAEC* with γδres-sacB-hyg-γδres cassette and the *M. smegmatis* Δ*uspAEC* mutant strain was confirmed by whole-genome sequencing of genomic DNA (MicrobesNG).

### Complementation of the *M. smegmatis* mutants

For the complementing plasmids either the *uspC* gene or the *uspAEC* operon were amplified by PCR (Q5 high-fidelity polymerase, NEB) from *M. smegmatis* or *Mtb* genomic DNA. The primers are shown in Table S1. The resulting PCR products were digested with EcoRI and HindIII and ligated into the integrative pMV361 plasmid, which contains the *hsp60* promoter, to generate the *Msmeg_uspC_pMV361*, *Mtb_uspC_pMV361* and *Msmeg_uspAEC_pMV361* plasmids. These plasmids were transformed into the *M. smegmatis* Δ*uspC* and Δ*uspAEC* mutants, respectively.

### Operon analysis

*M. smegmatis* and *M. bovis* BCG were grown to an OD_600_ of 0.8, guanidine thiocyanate (GTC) solution (1 M Tris-HCl pH 7.5, GTC (0.5% (w/v)), β-mercaptoethanol (1% (v/v)) was added and the pellets stored at −80 °C. 700 μL RLT buffer (Qiagen) containing 1% (v/v) β-mercaptoethanol was added to each sample and processed with bead beating (FastPrep® Lysing Matrix B tubes (FastPrep-24, MP Biomedics, Biospec)). Total RNA was isolated (RNeasy Mini kit (Qiagen)), including on-column DNase digestion with RNase-Free DNase. The RNA was treated with Turbo DNase (Turbo DNA-free kit, Ambion) to remove contaminating DNA, which was confirmed by PCR of *mysA* (MSMEG_2758). The isolated RNA was stored at −80 °C. The integrity of the RNA was checked by agarose gel electrophoresis and the amount and purity of RNA were assessed (Nanodrop). cDNA was synthesised using the Superscript IV Reverse Transcriptase (Invitrogen, UK) with random hexamer priming according to the manufacturer’s instructions (Invitrogen, 2015). Control cDNA samples were prepared through replacement of the Superscript IV Reverse Transcriptase with water to confirm the absence of genomic DNA (gDNA) contamination. Overlapping regions of the genes in the *uspABC* cluster were amplified from the cDNA using KAPA Taq polymerase (Kapa Biosystems, UK) and the primers indicated in Table S1. A positive control using species-specific mycobacterial gDNA was also included. The PCR products were analysed in a 1.2% agarose gel.

### Topology mapping

Topology mapping of transmembrane proteins was studied by using BlaTEM-1 (β-lactamase) as a periplasmic reporter, as previously described (McCann et al., 2007). Briefly, the *Mtb uspA, uspB, uspC* genes were amplified from gDNA by PCR and cloned into the pMZ101 vector to generate an *N*-terminal BlaTEM-1 fusion (a gift from Professor Stewart Cole, EPFL, Switzerland) or the pMZ101C vectors to generate a *C*-terminal fusion. The pMZ101c vector was generated by amplification of *blaTEM-1* from the pMZ101 vector and cloning into pMV261 cut with HindIII. This resulted in *uspA*_pMZ101, *uspB*_pMZ101, *uspC*_pMZ101 constructs in-frame with an *N-*terminal blaTEM-1 reporter and *uspA*_pMZ101c, *uspB*_pMZ101bc *uspC*_pMZ101c in-frame with a *C*-terminal blaTEM-1 reporter. Each construct was transformed into *M. smegmatis* PM759 (Δ*blaS1*) (a gift from Prof Martin Pavekla, University of Rochester, USA) and selected on Middlebrook 7H10 media supplemented with 40 μg/mL lysine, 25 μg/mL kanamycin and 50 μg/mL streptomycin. To perform topology mapping, the *M. smegmatis* strains were grown in LB broth supplemented with 40 μg/mL lysine, 25 μg/mL kanamycin and 0.05% Tween 80 to an OD_600_ of 0.8–1.0 and then spotted (10 μL) onto Middlebrook 7H10 media supplemented with 40 μg/mL lysine, 25 μg/mL kanamycin and 50 μg/mL streptomycin in the presence or absence of ampicillin (100 μg/mL), incubated for 3 days at 37 °C and the growth monitored. Three independent biological replicates were performed.

### Determination of minimum inhibitory concentrations

The minimum inhibitory concentrations (MIC) of all compounds were determined using the resazurin reduction microplate assay (REMA) as described previously (Palomino et al., 2002). *M. smegmatis* strains were grown to mid-log phase and the inoculum standardised to 1 × 10^6^ colony forming units (CFU)/mL before addition to the 96-well flat-bottom microtiter plate containing 2-fold serial dilutions of each drug in media. Rifampicin was also added to each plate as a control antibiotic and the last column of the plate was used as a control without the addition of compound. The plates were incubated without shaking for 24 h before addition of 25 μL resazurin (one tablet of resazurin (VWR) dissolved in 30 mL of sterile PBS). Following a further 24 h incubation at 37 °C the plates were assessed for colour development. The MIC values were determined as the lowest concentration of drug that prevented the colour change of resazurin (blue: no bacterial growth) to resofurin (pink: bacterial growth).

### Monitoring growth of *M. smegmatis*

*M. smegmatis* growth was monitored using a plate reader (Infinite F200Pro, Tecan Life Sciences) and the absorbance measured at 600 nm (OD_600_) at 37 °C for up to 96 h with orbital shaking at 180 rpm. The starting OD_600_ of the *M. smegmatis* was 0.05. The plates were sealed with parafilm and the perimeter wells were filled with sterile water (200 μL) to avoid evaporation. The curves were fitted to the data points using the Lowess fit in GraphPad Prism V8. Three biological replicates were performed for each condition.

### Phenotype Microarrays ™ (Biolog)

PM1 and PM2A Phenotype MicroArrays™ (Biolog Inc., Hayward CA, USA) were used to assess carbon usage phenotypes. *M. smegmatis* strains were grown to mid-logarithmic phase (OD_600_ = 0.6 – 0.8). To starve the cells the culture was pelleted by centrifugation (3,220 × g, 10 min, 22 °C), washed three times in PBS containing 0.05% tyloxapol (PBSTylo), resuspended in PBSTylo and incubated at 22 °C for 24 h. Following starvation, the cells were centrifuged (3,220 × g, 10 min, 22 °C), washed twice with PBSTylo and resuspended in IF-0a GN/GP to an OD_600_ of 0.68. The cells were added to the PM additive solutions with Dye mix G described in Table S2 resulting in an OD_600_ of 0.05. The PM plates were inoculated with the *M. smegmatis* strains (100 μL), sealed with double skin breathable film (4titude, Surrey, UK) and the plate lid sealed (microporous tape, Micropore™). The plates were transferred to a Tecan Freedom Evo 200 Robot coupled with Tecan Infinite F200Pro plate reader and incubated at 37 °C for 96 h with shaking at 180 rpm. Dye reduction was monitored at 595 nm every hour over 96 h. The data for each run were exported as ASCII files and collated into a single file using Python script (https://github.com/ConorEd/PlateCollater). The data was analysed in MATLAB with a short script for background deduction. Heatmaps with hierarchical clustering were produced using the “pheatmap” package (Kolde, 2013).

### Proteomics

#### Preparation of samples for proteomics

*M. smegmatis* strains (WT, Δ*uspC* and Δ*uspAEC,* 30 mL) were grown to an OD_600_ of 0.8 in 7H9 media. The cells were harvested (3,550 *x g*, 20 min, 4 °C), washed (3 × PBST) and the pellet resuspended in lysis buffer (PBS, 1 mM DTT, 1 mg/mL lysozyme, protease inhibitor (Pierce) pH 7.4) for 2 h at room temperature. 0.1 mm silica glass beads were then added and the cells were disrupted by bead-beating (4 × 45 secs on, 45 secs off and placed on ice between cycles, 6 m/sec, FastPrep-24 5G (MP Biomedicals)) followed by sonication (water sonicator bath) at room temperature for 15 min. The samples were centrifuged (2,300 *× g*, 20 min, 4 °C) and the supernatant collected. The protein concentration was determined by Qubit™ fluorometer (Invitrogen) using Qubit™ Protein Assay Kit (Invitrogen), according to the manufacturer’s protocol.

#### Proteomic analysis

Protein samples (15 µL) were mixed with 2x SDS loading dye and loaded directly onto the SDS-gel (BioRad AnykD Mini-PROTEAN TGX) and run for 5 min. The gel bands were cut from the gel and prepared for proteomics analysis as described previously (Li et al., 2019). In brief, samples were reduced with 10 mM tris-2(-carboxyethyl)-phosphine (TCEP), alkylated with 40 mM chloroacetamide (CAA) and then in-gel digested with trypsin (2.5 ng/mL) and the peptides extracted with 25% acetonitrile containing 5% formic acid. The extracted peptides were dried under vacuum to a volume of 20 μL and resuspended to a total volume of 50 μL in 2% acetonitrile, 0.1% trifluoroacetic acid. Mass Spectrometry was performed on a Thermo Orbitrap Fusion (Thermo Scientific) coupled to an Ultimate 3000 RSLCnano HPLC (Dionex) using an Acclaim PepMap µ-precolumn cartridge (300 µm i.d. × 5 mm, 5 μm, 100 Å) and an analytical Acclaim PepMap RSLC column (75 µm i.d. × 50 cm, 2 µm, 100 Å, Thermo Scientific). Mobile phase buffer A was composed of 0.1% formic acid in H_2_O and mobile phase B was composed of acetonitrile containing 0.1% formic acid. The gradient was programmed as follows: 4% B increased to 25% B over 90 min, then further increased to 35% B over 13 min, followed by 3 min 90% B with a flow rate of 250 nL/min. Survey scans of peptide precursors from 375 to 1575 *m*/*z* were performed at 120 K resolution (at 200 *m*/*z*) with a 2x10^5^ ion count target. The maximum injection time was set to 150 ms. Tandem MS was performed by isolation at 1.2 Th using the quadrupole, HCD fragmentation with normalised collision energy of 33, and rapid scan MS analysis in the ion trap. The MS^2^ ion count target was set to 3x10^3^ and maximum injection time was 200 ms. Precursors with charge state 2–6 were selected and sampled for MS^2^. The dynamic exclusion duration was set to 45 s with a 10 ppm tolerance around the selected precursor and its isotopes. Monoisotopic precursor selection was turned on and instrument was run in top speed mode.

#### Data analysis, data processing and annotation

The raw data were searched using MaxQuant with an integrated Andromeda search engine (V1.5.5.1) (Cox et al., 2011) against both the *M. smegmatis* database and the common contaminant database from MaxQuant. Peptides were generated from a tryptic digestion with up to two missed cleavages, carbamidomethylation of cysteines as fixed modifications, and oxidation of methionines as variable modifications. Precursor mass tolerance was 10 ppm and product ions were searched at 0.8 Da tolerances. For protein quantification, label free quantification (LFQ) was selected and proteins with LFQ minimum ratio count of 2 were retained. The PSM FDR, protein FDR and site decoy fraction were set to 1 for further analysis in Scaffold or to 0.01 for analysis in Perseus. Scaffold (version 4.6.2) was used to validate MS/MS based peptide and protein identifications. Peptide identifications were accepted if they could be established at greater than 95.0% probability by the Scaffold Local FDR algorithm. Protein identifications were accepted if they could be established at greater than 95.0% probability and contained at least 2 identified peptides. Proteins that contained similar peptides and could not be differentiated based on MS/MS analysis alone were grouped to satisfy the principles of parsimony. Proteins sharing significant peptide evidence were grouped into cluster. Data processing and annotation was performed used the Perseus module of MaxQuant version 1.6.2.2 (Tyanova et al., 2016). First, the reverse and contaminant hits (as defined in MaxQuant) were eliminated from the MaxQuant output files. Only protein groups identified with at least two uniquely assigned peptide and quantified with a minimum of two ratio counts were used for the analysis. For each experiment, the label free quantification intensity (LFQ) were transformed using the binary logarithm (log_2_). Protein groups were considered reproducibly quantified if identified and quantified in at least two replicates, missing LFQ intensity scores were assigned from a normal distribution. Protein groups were assigned a probability value (p-value) using a two-sample Student’s T-Test. p-values were subject to a -log_10_ transformation. Proteins were considered significant if the p-value < 0.05 (-log_10_(p-value) greater than 1.30) and had a two-fold change in protein expression (log_2_(LFQ difference) greater than 1 or < -1). Protein function, product, functional category were assigned based on Mycobrowser (release 3) annotations (Kapopoulou et al., 2011).

## Results

### Characterisation of the *uspABC* operon in *M. smegmatis* and *M. bovis* BCG

**Fig. 1:**
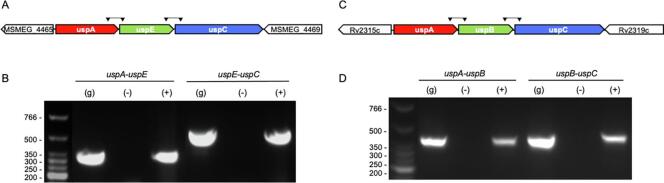
*uspABC* operon analysis for *Mycobacterium smegmatis (*A + B*)* and *Mycobacterium bovis* BCG (C + D) A) Schematic representation of the *M. smegmatis* gene cluster and adjacent genes. The locations of the primers used are highlighted. B) RT‐PCR analysis of genes contained within the *M. smegmatis uspAEC* putative operon. C) Schematic representation of the *M. bovis* BCG gene cluster, which is identical to the region found in *Mtb*. The locations of the primers used are highlighted. D) RT‐PCR analysis of genes contained within the *M. bovis* BCG *uspABC* putative operon. (g) genomic DNA, (+) cDNA, (-) reverse transcriptase negative control. The primer pairs are shown with black triangles.

To determine if the *uspA-uspB-uspC* genes are co-transcribed, total RNA was extracted from either *M. smegmatis* or *M. bovis* BCG as a replacement for *Mtb* since comparison with the sequence of *uspABC* region in the *Mtb* genome identified that the intergenic regions in *M. bovis* BCG are identical to *Mtb* and show only two non-synonymous single nucleotide polymorphism (SNPs) changes in the *uspA* (nucleotide change g380c: amino acid V127L) and *uspC* (nucleotide change c1061g: amino acid L353V) coding sequences. To elucidate whether a single transcript was present the intergenic regions between the adjacent genes *uspA-uspB/E* and *uspB/E-uspC* (in *M. smegmatis uspB* is annotated as *uspE*) were amplified by reverse transcriptase PCR (RT-PCR). Successful amplification for the overlapping regions was observed, and no amplification was detected in controls without reverse transcriptase, confirming that the *uspA-uspB/E-uspC* genes function as a transcriptional unit and operon (Fig. 1).

### Topology mapping of *Mtb* UspA, UspB and UspC in *M. smegmatis*

**Fig. 2:**
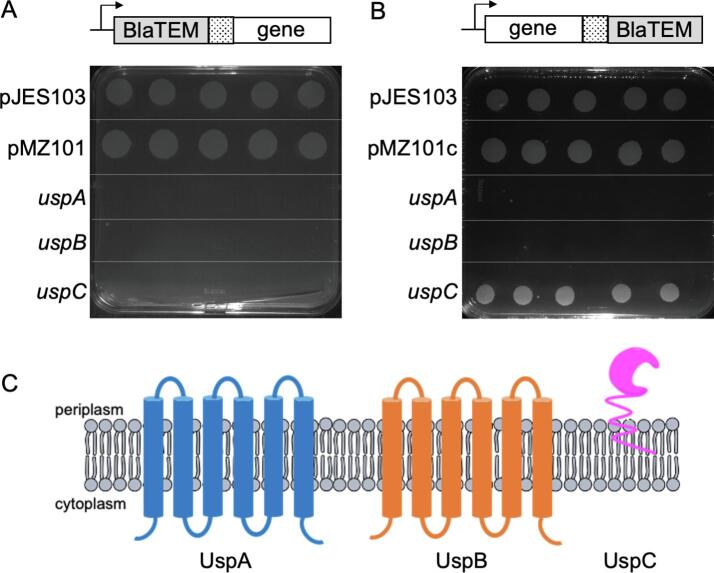
Topology mapping of the *Mtb* UspABC transporter. Schematic representation of the in-frame *uspA, uspB* and *uspC* BlaTEM-1 fusion constructs used in this study A) Growth of *M. smegmatis* PM759 transformed with the *N*-terminal blaTEM-*Mtb uspA, uspB* and *uspC* fusions on ampicillin. B) Growth of *M. smegmatis* PM759 transformed with the C-terminal blaTEM-*Mtb uspA, uspB* and *uspC* fusions on ampicillin. Each experiment was repeated three times with five replicates and the same results were obtained. The *Mtb mpt63-blaTEM* C-terminal fusion construct (pJES103) was included as a positive control as the Mpt63 protein is exported to the periplasm. C) Topology models of *Mtb* UspA, UspB and UspC determined from the BlaTEM-1 fusion data obtained (Fig. 2A and 2B).

Computational transmembrane analysis predicts that both UspA and UspB are integral membrane proteins with 6 transmembrane helices and that UspC has a putative transmembrane region at the *N*-terminus (Supplementary Fig. S2) (Sonnhammer et al., 1998). In order to confirm the *in* silico prediction and determine the transmembrane topology in intact mycobacterial cells, *Mtb uspA, Mtb uspB* and *Mtb uspC N-* and *C-* terminal fusions were generated to the truncated periplasmic β -lactamase reporter BlaTEM-1 as a selectable extracellular reporter (Fig. 2) (McCann et al., 2007, Perkowski et al., 2017). Each construct was transformed into a β -lactamase sensitive strain of *M. smegmatis* (PM759) to enable the selection of BlaTEM-fusions that were resistant to ampicillin (Flores et al., 2005). The recovery of clones selected on ampicillin indicates that BlaTEM is located extracellularly and no growth indicates that BlaTEM is located intracellularly. We observed no growth for all UspA and UspB BlaTEM-fusions (Fig. 2) indicating that the *N*- and *C*-termini for both proteins are located in the cytoplasm. In contrast, our topology model based on the fusion data of *Mtb* UspC places the *N-*terminus in the cytoplasm/buried within the membrane and the *C*-terminus is located within the periplasm (Fig. 2), which is the typical arrangement for substrate binding domains (Locher, 2016, Thomas et al., 2020).

### *M. smegmatis uspC and uspAEC* are dispensable for growth under standard culture conditions

To explore the essentiality of the *uspC* substrate-binding domain and *uspAEC* transporter, deletion mutants were generated in *M. smegmatis*. The Δ*uspC* mutant was generated using a two-step allelic exchange to introduce an unmarked deletion into *uspC* in *M. smegmatis* (Supplementary Fig. S3). We were not able to generate the Δ*uspAEC* mutant strain using this method, and instead used phage and recombineering to generate a marked Δ*uspAEC* deletion mutant, whereby *uspAEC* is replaced by a cassette containing *sacB* and hygromycin resistance genes (Supplementary Fig. S3) (Flores et al., 2005). The Δ*uspC* and Δ*uspAEC* mutant strains were confirmed by whole genome sequencing. The mutant strains were complemented by introducing a copy of either the *M. smegmatis* or *M. tuberculosis uspC* gene or the *M. smegmatis uspAEC* genes under the control of the *hsp60* promoter on an episomal plasmid (pMV361). Growth of the wild-type, Δ*uspC*, Δ*uspAEC* and complemented strains (Δ*uspC*:*Msmeg_uspC_pMV361,* Δ*uspC*:*Mtb_uspC_pMV361,* Δ*uspAEC*:*Msmeg_uspAEC_pMV361*) was monitored in Tryptic Soy Broth (TSB) for 24 h and in Sauton’s media for 96 h. The Δ*uspC* and Δ*uspAEC* strains exhibited growth rates similar to that of the wild type (WT) strain indicating that under these conditions *uspC* and *uspAEC* are not required for the *in vitro* growth of *M. smegmatis* (Supplementary Fig. S4). Loss of either *uspC* or *uspAEC* had no observable effect on the colony morphology of Δ*uspC* and Δ*uspAEC* mutant strains compared to the wild-type strain on LB agar (Supplementary Fig. S5).

### The UspAEC transporter does not significantly alter susceptibility of *M. smegmatis* to antibiotics

*Mycobacteria* have complex waxy cell envelopes, which render many antibiotics ineffective (Brennan, 2003, Brennan and Nikaido, 1995). It is therefore possible that existing anti-tubercular agents hijack endogenous transporters to gain access to their intracellular targets (Fullam and Young, 2021). Consequently, we wanted to evaluate the accessibility and susceptibility of the Δ*uspC* and Δ*uspAEC* transporter mutant strains to a panel of antibiotics with different mechanisms of action, including the aminoglycosides (apramycin, gentamycin, kanamycin, neomycin, streptomycin, spectinomycin), β -lactams (ampicillin), carbapenems (meropenem), fluoroquinolones (nalidixic acid), glycopeptides (vancomycin), chloramphenicol, tetracycline, and the antimycobacterial agent rifampicin using the resazurin-based reduction assay (Palomino et al., 2002). The Δ*uspC* and Δ*uspAEC* mutants and WT strain displayed similar MICs for all antibiotics tested (Table 1). Small changes were observed for the aminoglycoside spectinomycin, which resulted in ~ 2-fold increase in the MIC, and for vancomycin, a peptidoglycan cell wall inhibitor, which resulted in increased sensitivity (~ 2-fold) of the Δ*uspC* and Δ*uspAEC* mutants compared to the wild-type strain.

**Table 1:** MIC values (µg/mL) of *M. smegmatis and the* Δ*uspC* and Δ*uspAEC* mutant strains.

Antibiotic	WT	Δ*uspC*	Δ*uspAEC*
Apramycin	1.56–3.13	3.13	3.13
Gentamycin	6.25–12.50	12.50	12.50
Kanamycin	0.39–0.78	0.39–0.78	0.39–0.78
Neomycin	3.13	3.13	3.13
Spectinomycin	37.50–75	75–150	150
Streptomycin	0.20	0.20	0.20–0.39
Ampicillin	150	150	150
Meropenem	1.56–6.25	3.13–6.25	3.13–6.25
Nalidixic Acid	150	75–150	150
Vancomycin	1.56	0.78–1.56	0.78
Chloramphenicol	25	25	25
Tetracycline	0.39–0.78	0.39–0.78	0.78–1.56
Rifampicin	2.50–5	2.50–5	2.50–5

### Difference in carbon substrate usage between the Δ*uspC* and Δ*uspAEC* mutants and wild-type *M. smegmatis*

**Fig. 3:**
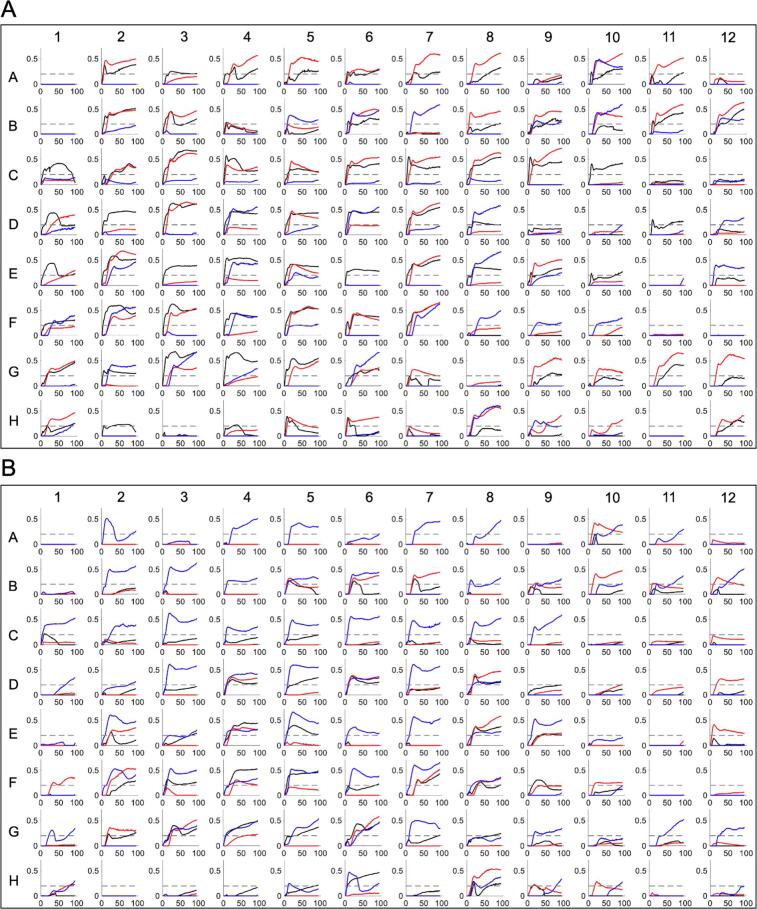
Carbon source utilisation profiles of *M. smegmatis*, *M. smegmatis* Δ*uspC* and *M. smegmatis* Δ*uspAEC* mutants. XY plot of *M. smegmatis* WT (black), *M. smegmatis* Δ*uspC* (red) and Δ*uspAEC* (blue) grown on Biolog Phenotype MicroArray^TM^ PM01 (A) and PM02A (B) plates. x-axis is time in hours and y-axis is respiration signal. The respiration signal is used as a read-out for bacterial growth. The individual carbon source plate maps are shown in Supplementary Fig. S6. (For interpretation of the references to colour in this figure legend, the reader is referred to the web version of this article.)

**Fig. 4:**
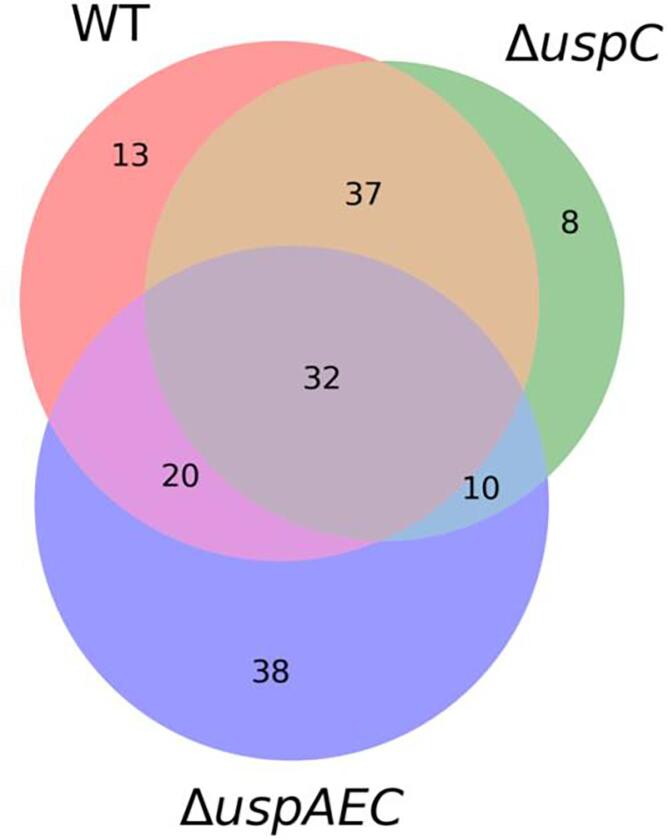
Comparison of the carbon source utilisation profiles of *M. smegmatis*, *M. smegmatis* Δ*uspC* and *M. smegmatis* Δ*uspAEC*. Total number of carbon sources which resulted in a substantial redox dye reduction (respiration signal ≥ 0.2) by *M. smegmatis* WT, *M. smegmatis* Δ*uspC* and *M. smegmatis* Δ*uspAEC*. The full list and identity of each carbon source is in Supplementary List S1.

To evaluate the carbon substrate utilisation profiles of the Δ*uspC* and Δ*uspAEC* mutants we assessed the phenotypic growth of these strains in the presence of 190 different single carbon sources using commercial Phenotype Microarrays^TM^ (Biolog) and compared these to the growth of wild-type *M. smegmatis* under the same experimental conditions. In each plate a negative control that lacked a carbon source was included (well A1). The kinetics of the dye reduction values for each strain over 96 h with the defined carbon source are shown in Fig. 3. Overall substantial dye reduction (signal ≥ 0.2), indicating that the strain was able to grow in the presence of an individual carbon source, was observed for 102 single carbon sources for *M. smegmatis*, 87 carbon sources for the Δ*uspC* mutant strain and 100 carbon sources for the Δ*uspAEC* mutant over 96 h (Fig. 3, Supplementary List S1). The identities of the carbon sources are listed in Supplementary List S1. Comparison of the unique carbon sources used by each mutant reveals that 8 unique carbon sources are used by Δ*uspC* and 38 unique carbon sources are used by the Δ*uspAEC* mutant (Fig. 4 and Supplementary Table S1) that are not used by the wild-type strain.

**Fig. 5:**
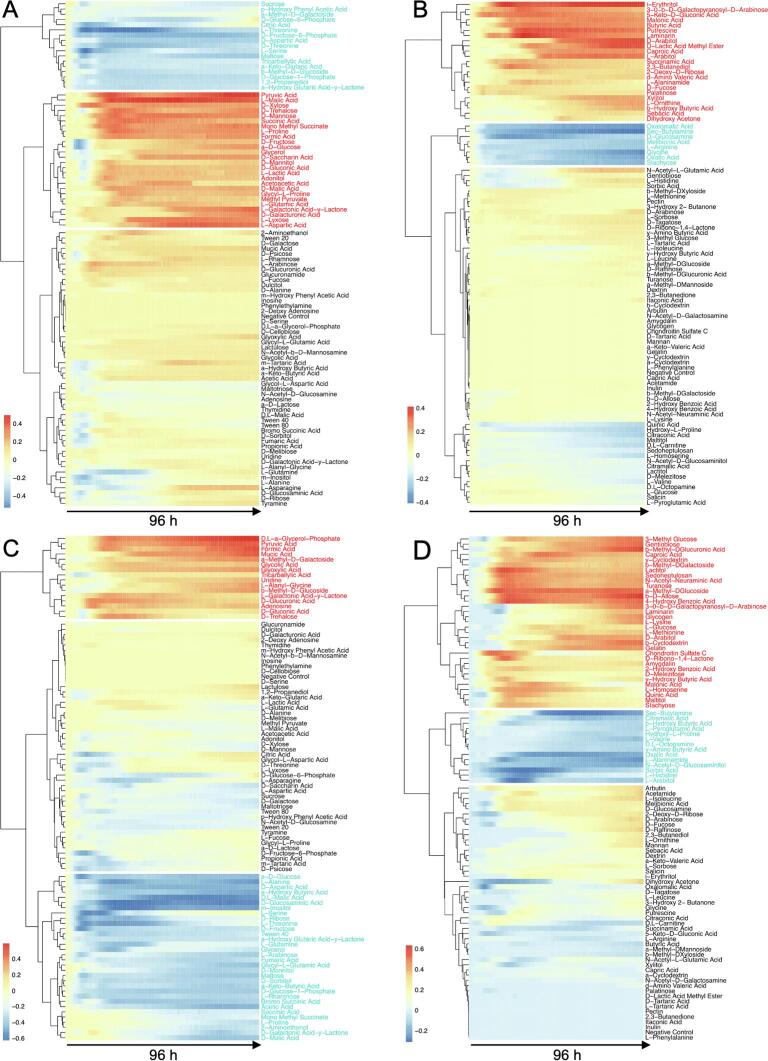
Heat map and hierarchical clustering depending on the carbon substrate utilisation of Δ*uspC* and Δ*uspAEC* mutant strains grown in Phenotype Microarray (Biolog) plates PM01 and PM02A*.* A + B: Δ*uspC vs M. smegmatis* WT (A: PM01, B: PM02A). C + D: Δ*uspAEC vs M. smegmatis* WT (C: PM01, D: PM02A). Red represents high substrate utilisation and blue represents low substrate utilisation compared to WT *M. smegmatis* over 96 h. Carbon sources in red text indicated increased growth and carbon sources in blue font indicate reduced growth of the mutant strains compared to WT. (For interpretation of the references to colour in this figure legend, the reader is referred to the web version of this article.)

To further evaluate the differences in growth phenotypes between the mutant strain and wild-type *M. smegmatis* at the same time-point over 96 h, heat maps were generated from the calculated differences in the dye reduction values (Fig. 5). In the heat maps a positive dye reduction difference between the mutant strain compared to wild-type is shown in red and indicates that the mutant grows better on the individual carbon source. In contrast, a negative dye reduction difference between the mutant strains compared to wild-type is shown in blue indicating that the mutant grew less than wild-type.

**Fig. 6:**
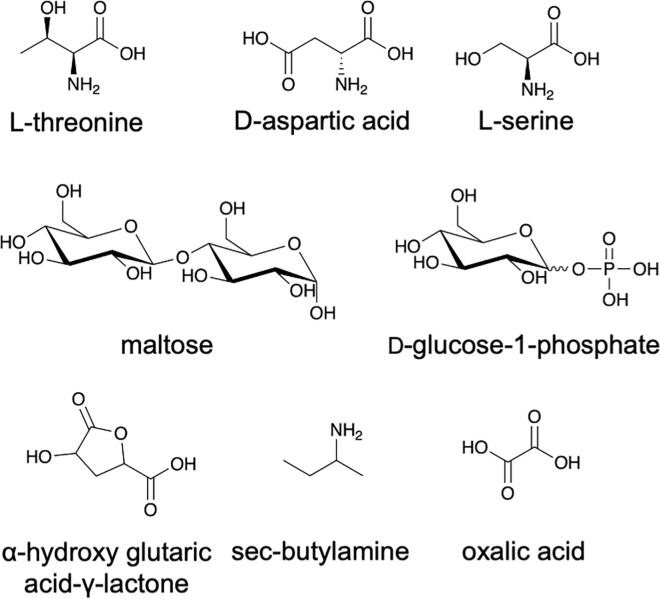
Carbon sources that are utilised less by the Δ*uspC* and Δ*uspAEC* mutant strains compared to wild-type *M. smegmatis.*

Heat map analysis revealed that there were 39 different carbon sources and 10 identical carbon sources (pyruvic acid, formic acid, caproic acid, malonic acid, L-Galactonic Acid-y-Lactone, D-gluconic acid, D-trehalose, 3-O- β -D-galactopyranosyl-D-arabinose, laminarin and D-arabitol) that resulted in the growth promotion of Δ*uspC* and also Δ*uspAEC* mutant strains compared to wild-type *M. smegmatis* (Supplementary List S2*)*. The utilisation of these alternative carbon sources could be a result of compensatory changes in cell metabolism in the absence of the UspAEC transporter. We were particularly interested in the identification of carbon sources with a decreased dye signal as this may indicate that the mycobacterial UspAEC transporter plays a role in the uptake of these substrates. In this study we observed that 25 carbon sources for Δ*uspC* and 46 carbon sources for Δ*uspAEC* result in reduced growth of these knock-out strains compared to wild-type *M. smegmatis* (Fig. 5, Supplementary List S3). Overall, a range of acids, sugars and amino acids were identified as carbon sources that result in decreased growth of the mutant strains and the identity of the carbon sources are listed in Supplementary List S3. Eight carbon sources resulted in reduced growth for both strains: L-threonine, D-aspartic acid, L-serine, maltose, D-glucose-1-phosphate, α-hydroxy glutaric acid-γ-lactone, *sec*-butylamine, and oxalic acid (Fig. 6). We also identified a number of carbon sources that were only able to support the respiration of either the Δ*uspC* or the Δ*uspAEC* mutant. Four substrates: α-methyl-D-galactoside, tricarballylic acid, β-methyl-D-glucoside and stachyose, gave a positive dye reduction signal for the Δ*uspAEC* mutant and a negative dye reduction signal for the Δ*uspC* mutant. In contrast, eleven substrates: α-D-glucose, D-fructose, glycerol, D-mannitol, succinic acid, mono methyl succinate, L-proline, D-malic acid, β-hydroxy butyric acid, L-alaninamide and L-arabitol, gave a positive dye reduction signal for the Δ*uspC* mutant and a negative dye reduction signal for the Δ*uspAEC* mutant. Interestingly we found that glucosamine did not support the growth of the Δ*uspC M. smegmatis* mutant. This finding is in agreement with our previous biochemical studies that found *Mtb* UspC binds to and recognises glucosamine and other amino sugars (Fullam et al., 2016). The reasons for the distinct carbon source utilisation profiles between the two mutant strains is unclear. However, these results suggest that there are important differences in the characteristics of *M. smegmatis* strains that lack the UspC substrate binding domain compared to the entire UspAEC ABC-transporter.

### Proteomic pathway mapping

**Fig. 7:**
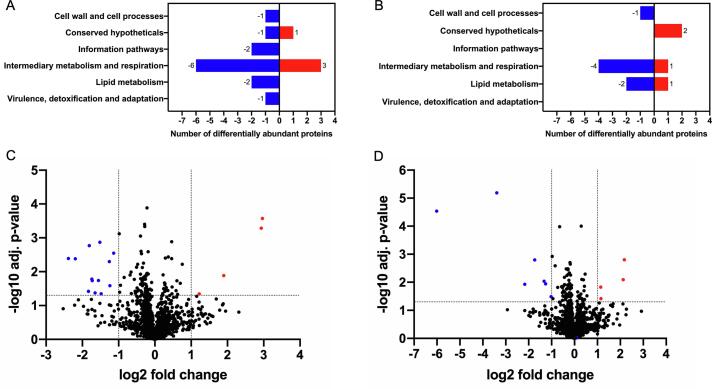
Proteomics analysis of *M. smegmatis* Δ*uspC* and Δ*uspAEC* mutants A + B): Δ*uspC, C + D):* Δ*uspAEC*. A) Proteins that are in higher (red) or lower (blue) abundance in Δ*uspC* compare to wild-type *M. smegmatis* by functional category. B) Proteins that are in higher (red) or lower (blue) abundance in Δ*uspAEC* compare to wild-type *M. smegmatis* by functional category. C) Volcano plot of the obtained proteomics datasets, determining the fold-change of differentially expressed proteins between the Δ*uspC* mutant and WT strain. Downregulated (blue), upregulated (red). Adjusted p-value ≤ 0.05 and log_2_-fold-change ≥±1. D) Volcano plot of the obtained proteomics datasets, determining the fold-change of differentially expressed proteins between the Δ*uspAEC* mutant and WT strain. Downregulated (blue), upregulated (red). Adjusted p-value ≤ 0.05 and log_2_-fold-change ≥±1. (For interpretation of the references to colour in this figure legend, the reader is referred to the web version of this article.)

To identify pathways that are modulated in strains of *M. smegmatis* that lack functional components of the UspAEC ABC-transporter we profiled the proteomes of the Δ*uspC* and Δ*uspAEC* mutants in nutrient rich broth (7H9) and compared these to that of the wild-type strain. The isolated proteins were identified by mass spectrometry and quantification of the identified proteins was determined using MaxQuant (Cox and Mann, 2008). The total number of proteins that had at least two unique peptides in at least two replicates identified in *M. smegmatis* WT was 1,622, in *M. smegmatis* Δ*uspC* was 1,577 and in *M. smegmatis* Δ*uspAEC* was 1,589. To determine the changes in protein levels between *M. smegmatis* WT, Δ*uspC* and Δ*uspAEC* quantification of the identified proteins was determined by MaxLFQ (label-free quantification, MaxQuant) resulting in 1,383 proteins for all strains. The complete list of proteins detected is in Supplementary List S4. Comparative proteomic analysis identified 17 differently abundant proteins in the Δ*uspC* strain and 11 differentially abundant proteins in the Δ*uspAEC* strain (adjusted p-value ≤ 0.05 and log_2_-fold-change ≥±1) versus wild-type *M. smegmatis.* Of these, 4 proteins were enriched and 13 proteins were at lower abundance in the Δ*uspC* mutant and 4 proteins were enriched and 7 proteins were at lower abundance in the Δ*uspAEC* strain, Fig. 7. The identities of these proteins and their relative abundance are summarised in Table 2, Table 3 and Supplementary List S4.

**Table 2:** Differentially abundant proteins in Δ*uspC.*

Gene name	**Rv** homologue	Product	Functional category	Log_2_FC
MSMEG_4916	Rv1327c (*glgE*)	α −1,4-glucan:maltose-1-phosphate maltosyltransferase	Intermediary metabolism and respiration	3.0
MSMEG_1392		alcohol dehydrogenase, class IV	Intermediary metabolism and respiration	2.9
MSMEG_0684		aldehyde oxidase and xanthine dehydrogenase, molybdopterin binding	Intermediary metabolism and respiration	1.9
MSMEG_5634	Rv0910	conserved hypothetical	Conserved hypothetical	1.2
MSMEG_0366		hypothetical protein	Conserved hypotheticals	−2.4
MSMEG_1424	Rv0694	FMN-dependent dehydrogenase, possible L-lactate dehydrogenase	Intermediary metabolism and respiration	−2.2
MSMEG_1594		enoyl-CoA hydratase	Lipid metabolism	−1.8
MSMEG_2433	Rv2911 (*dacB2*)	D-alanyl-D-alanine carboxypeptidase	Cell wall and cell processes	−1.8
MSMEG_0889		succinic semialdehyde dehydrogenase	Intermediary metabolism and respiration	−1.7
MSMEG_5685	Rv0883c	DNA-binding protein	Information pathways	−1.7
MSMEG_4976		isochorismatase hydrolase	Intermediary metabolism and respiration	−1.6
MSMEG_1410	Rv0687	carveol dehydrogenase	Intermediary metabolism and respiration	−1.6
MSMEG_5119 (*pruA*)	Rv1187 (*rocA*)	1-pyrroline-5-carboxylate dehydrogenase	Intermediary metabolism and respiration	−1.5
MSMEG_5073	Rv1220c	O-methyltransferase, family protein 3	Intermediary metabolism and respiration	−1.5
MSMEG_1952	Rv3198c	ATP-dependent DNA helicase	Information pathways	−1.3
MSMEG_1940	Rv3203	hydrolase, alpha/beta fold family protein	Lipid metabolism	−1.2
MSMEG_3962		lactate 2-monooxygenase	Virulence, detoxification and adaptation	−1.1

**Table 3:** Differentially abundant proteins in Δ*uspAEC.*

Gene name	Rv homologue	Product	Functional category	Log_2_FC
MSMEG_1392		alcohol dehydrogenase, class IV	Intermediary metabolism and respiration	2.2
MSMEG_6365	Rv3780	conserved hypothetical protein	Conserved hypotheticals	2.1
MSMEG_5915	Rv3516 (*echA19*)	enoyl-CoA hydratase	Lipid metabolism	1.2
MSMEG_1530		integral membrane protein	Conserved hypotheticals	1.1
MSMEG_0408 (*pks*)		type I modular polyketide synthase	Lipid metabolism	−6.0
MSMEG_5866	Rv0761c (*adhB*)	alcohol dehydrogenase B	Intermediary metabolism and respiration	−3.4
MSMEG_6519		pyridoxamine 5′-phosphate oxidase family protein	Intermediary metabolism and respiration	−2.2
MSMEG_5906	Rv3504 (*fadE26*)	putative acyl-CoA dehydrogenase	Intermediary metabolism and respiration	−1.7
MSMEG_5594		ferredoxin-dependent glutamate synthase	Intermediary metabolism and respiration	−1.3
MSMEG_0641		binding-protein-dependent transport systems inner membrane component	Cell wall and cell processes	−1.3
MSMEG_0861 (*psd*)	Rv0437c	phosphatidylserine decarboxylase	Lipid metabolism	−1.0

## Discussion

As drug resistant strains of *Mtb* escalate and it becomes increasingly difficult to treat these emerging TB cases in the clinic, it is paramount that innovative, next-generation TB drugs and diagnostics are developed. One reason that a significant number of antibiotics are ineffective against the *Mtb* pathogen is that the highly waxy, impermeable mycobacterial cell envelope prevents these compounds from reaching their intracellular target, which significantly limits their effectiveness (Brennan, 2003, Brennan and Nikaido, 1995, Abrahams and Besra, 2021, Batt et al., 2020). One potential strategy to circumvent the bottleneck in TB drug discovery efforts is to hijack endogenous mycobacterial transporters to improve the uptake of anti-tubercular agents (Fullam and Young, 2021). However, to exploit these import systems, we first need to determine the physiological role of the mycobacterial transporters and map their substrate recognition profiles. In this work we have started to characterise the role of the UspABC carbohydrate ABC-transporter, which is highly conserved across mycobacterial species, and is defined as a core gene set in the leprosy bacillus that has undergone significant genome decay (Cole et al., 2001).

In order to characterise the role of UspABC in mycobacteria we first investigated whether the *uspABC* genes are co-transcribed by RT-PCR analysis. We found that the transcripts from the intergenic regions between the genes of *uspA-uspB/E* and *uspB/E-uspC* were amplified, indicating that in *M. bovis* BCG and *M. smegmatis uspA-uspB/E-uspC* are functionally expressed and belong to a single transcriptional unit. This operon structure is typical for ABC-transporters, although there are examples where the genes that encode for the transporter do not cluster together (Tomii and Kanehisa, 1998). Next, we wanted to determine the location and orientation of the *N*- and *C-*termini of the UspAB membrane spanning domains because there is a link between membrane protein topology and function (Von Heijne, 2006). The *Mtb* UspA- and UspB-BlaTEM1 fusions indicate a N_in_-C_in_ orientation for these 6-transmembrane helical integral transport proteins, which is the predominant organisation of membrane proteins in prokaryotic cells (Von Heijne, 2006). As expected, the associated substrate recognition domain is located in the periplasm with the *N-*terminus of UspC anchored to the membrane. Overall, our experimentally derived topology maps indicate that *Mtb* UspABC conforms to the typical arrangement of ABC-transporters that has been determined from resolving high-resolution structures (Locher, 2016, Thomas et al., 2020, Von Heijne, 2006).

To investigate the endogenous role of UspABC in mycobacteria, we deleted the *uspC* and *uspAEC* genes from *M. smegmatis* and investigated the phenotype using a number of approaches. Deletion of either *uspC* or *uspAEC* resulted in no obvious changes in morphologies or growth rates of the mutant strains compared to wild-type, indicating that in *M. smegmatis* the UspAEC transporter is not essential *in vitro*. *M. smegmatis* is a non-pathogenic soil dwelling bacteria often used as a model organism for *Mtb* that has a significantly higher number of putative ABC-transporters for the import of carbohydrates compared to *Mtb*, 28 *vs* 4 respectively, which likely reflects the different environmental niches and lifestyles of these mycobacterial species (Niederweis, 2008, Braibant et al., 2000). A number of studies have found that each *Mtb* carbohydrate transporter is highly specific for a defined substrate (Fenn et al., 2019, Fullam et al., 2016, Furze et al., 2021), and further experiments are now underway to investigate the role of UspABC in *Mtb*.

The intracellular targets of the front-line anti-tubercular agents are well established; however, it remains unclear whether these antibiotics are actively transported into the *Mtb* cell. To ascertain whether *M. smegmatis* UspAEC is involved in the uptake of antibiotics we conducted MIC testing against the Δ*uspC* and Δ*uspAEC* mutants. From this, we have deduced that the *M. smegmatis* UspAEC transporter is not the main route of import for the antibiotics tested. Vancomycin, a glycopeptide which interacts with cell wall peptidoglycan motifs, resulted in ~ 2-fold reduction in the MIC of both mutant strains, suggesting that strains of *M. smegmatis* that lack a functional UspABC transporter result in subtle changes in the composition of the mycobacterial cell wall. Our proteomics study also provided further evidence that alterations in cell wall peptidoglycan may occur since we found that Dacb2 (MSMEG_2433), a D-alanyl-D-alanine carboxypeptidase involved in removing the terminal D-alanine residue from the pentapeptide side chains (Baranowski et al., 2018), is down-regulated in the Δ*uspC* strain. These results link with our previous studies we found that the *Mtb* UspC substrate binding protein recognises amino-sugars and the hypothesis that this transporter has a possible role in recycling peptidoglycan fragments.

Biolog Phenotypic microarray technology has been used a tool to determine the metabolic activity under defined growth conditions and enables gene function to be correlated with the observed phenotype (Khatri et al., 2013, Baloni et al., 2014, Bochner, 2009). In this study we used this technology to profile the carbon source utilisation patterns of Δ*uspC* and Δ*uspAEC* mutants. We had postulated that the Δ*uspC* and Δ*uspAEC* mutant strains would have similar profiles since in order for transport to occur the transporter substrate must first be recognised by the substrate binding domain (UspC) before being translocated by the transmembrane components (UspAB). Instead, we observed distinct differences in the substrate utilisation and metabolic flexibility of these two strains. It is possible that the UspABC transporter still functions after removal of the UspC substrate domain, which is facilitated through its replacement with a solute-binding protein from another ABC-transporter. This ‘swapping’ of substrate binding domains has been proposed to occur for carbohydrate ABC-transporters in *Mycobacterium avium* and is likely to result in an altered substrate uptake profile of the transporter (Lamont et al., 2013). In this study we were particularly interested in the identification of single carbon sources that supported the growth of wild-type *M. smegmatis* but were not utilised by the Δ*uspC* and Δ*uspAEC* mutant strains as a route to defining the substrate profile of this transporter. From this study we found 8 substrates were utilised to a lesser extent by Δ*uspC* and Δ*uspAEC* and range in chemical diversity, including amino acids, carboxylic acids and sugars (Fig. 6). The identification that neither mutant strain can utilise D-glucose-1-phosphate is of particular interest as we previously found from biochemical analyses that *Mtb* UspC has a preference for phosphorylated amino-sugars (Fullam et al., 2016) indicating that this substrate, or analogues of this substrate, may be important. Furthermore, we also found that Δ*uspC* was not able to grow on glucosamine, further corroborating our earlier findings (Fullam et al., 2016). Taken together, these findings suggest that the mycobacterial UspABC transporter has a physiological role in the uptake of amino-sugars *in vitro,* and that this recycling process may influence peptidoglycan biosynthesis.

The comparative proteomic analysis provides a description of the proteins that differ between the Δ*uspC* and Δ*uspAEC* mutants compared to wild-type *M. smegmatis* at a single time-point. Similar to the data obtained from the phenotypic microarrays we found that the proteomic profiles also differed between Δ*uspC* and Δ*uspAEC*. In this study, only one protein was found in higher abundance in both mutants: MSMEG_1392, an alcohol dehydrogenase belonging to class IV. We also observed an ~ 8-fold increase in the abundance of the maltosyltransferase GlgE (MSMEG_4916) in Δ*uspC* and ~ 3.6-fold increase in Δ*uspABC.* GlgE is essential in *Mtb* and uses maltose-1-phosphate (maltose-1P) donor to synthesise α-glucan (Kalscheuer et al., 2010a). It is interesting to note that in our phenotypic microarray data we found that both Δ*uspC* and Δ*uspABC* mutants were unable to use maltose suggesting a link between the UspABC transporter and the utilisation of intracellular glycogen when nutrient availability is limited. Although UspABC appears to be predominantly involved in the uptake of carbohydrates, we identified alterations in the abundance of proteins involved in lipid metabolism: FadE26, EchA19 and Pks. It is possible that in mycobacteria there is an important interplay between carbohydrate and lipid metabolism under conditions such as starvation.

A thorough understanding of the nutrient requirements of *Mtb* and the transport processes involved in their uptake could provide important insights into the intracellular survival mechanisms of the *Mtb* pathogen and provide a route to the rational design of novel therapeutics and diagnostics that specifically target these import systems. Overall, our studies highlight that the *M. smegmatis* UspAEC has role in the uptake of phosphorylated sugars and links transporter function with alterations in pathways involved in carbohydrate and lipid metabolism. Whether mycobacterial strains that lack a functioning UspABC transporter resulting in alterations in carbohydrate substrate utilisation affect the composition of the *Mtb* cell envelope is an important question that will be defined in our future studies.

## CRediT authorship contribution statement

**Magdalena Karlikowska:** Conceptualization, Formal analysis, Investigation, Methodology, Writing - original draft, Writing - review & editing. **Albel Singh:** Investigation, Methodology, Writing - original draft, Writing - review & editing. **Apoorva Bhatt:** Investigation, Methodology, Supervision. **Sascha Ott:** Formal analysis, Investigation, Methodology. **Andrew R. Bottrill:** Formal analysis, Investigation, Methodology, Writing - original draft, Writing - review & editing. **Gurdyal S. Besra:** Investigation, Methodology, Supervision, Writing - original draft, Writing - review & editing. **Elizabeth Fullam:** Conceptualization, Formal analysis, Funding acquisition, Investigation, Methodology, Supervision, Writing - original draft, Writing - review & editing.

## Declaration of Competing Interest

The authors declare that they have no known competing financial interests or personal relationships that could have appeared to influence the work reported in this paper.
