# Supplementary material for: Biochemical and phenotypic characterisation of the Mycobacterium smegmatis transporter UspABC
Source: Cell Surf. 2021 Apr 24;7:100052. doi: 10.1016/j.tcsw.2021.100052 (PMC8281650; doi:10.1016/j.tcsw.2021.100052)
Supplement: Supplementary data 1 [file mmc1.pdf]

Supplementary Material

Biochemical and phenotypic characterisation of the *Mycobacterium smegmatis* transporter UspABC

Magdalena Karlikowska[1], Albel Singh[2], Apoorva Bhatt[2], Sascha Ott[3, 4], Andrew R Bottrill[1], Gurdyal S Besra[2], Elizabeth Fullam[1]*

[1] School of Life Sciences, University of Warwick, Coventry, CV4 7AL, UK
[2] Institute of Microbiology & Infection, School of Biosciences, University of Birmingham, Birmingham, B15 2TT, UK
[3] Warwick Medical School, University of Warwick, CV4 7AL, UK
[4] Bioinformatics Research Technology Platform, University of Warwick, Coventry, CV4 7AL

*Email for correspondence: e.fullam@warwick.ac.uk; (Tel. +44 (0)2476 574239)

SI Fig. S1. Genetic organisation of *uspABC* in mycobacterial species. Genes are colour coded according to G+C content, adapted from Xbase.

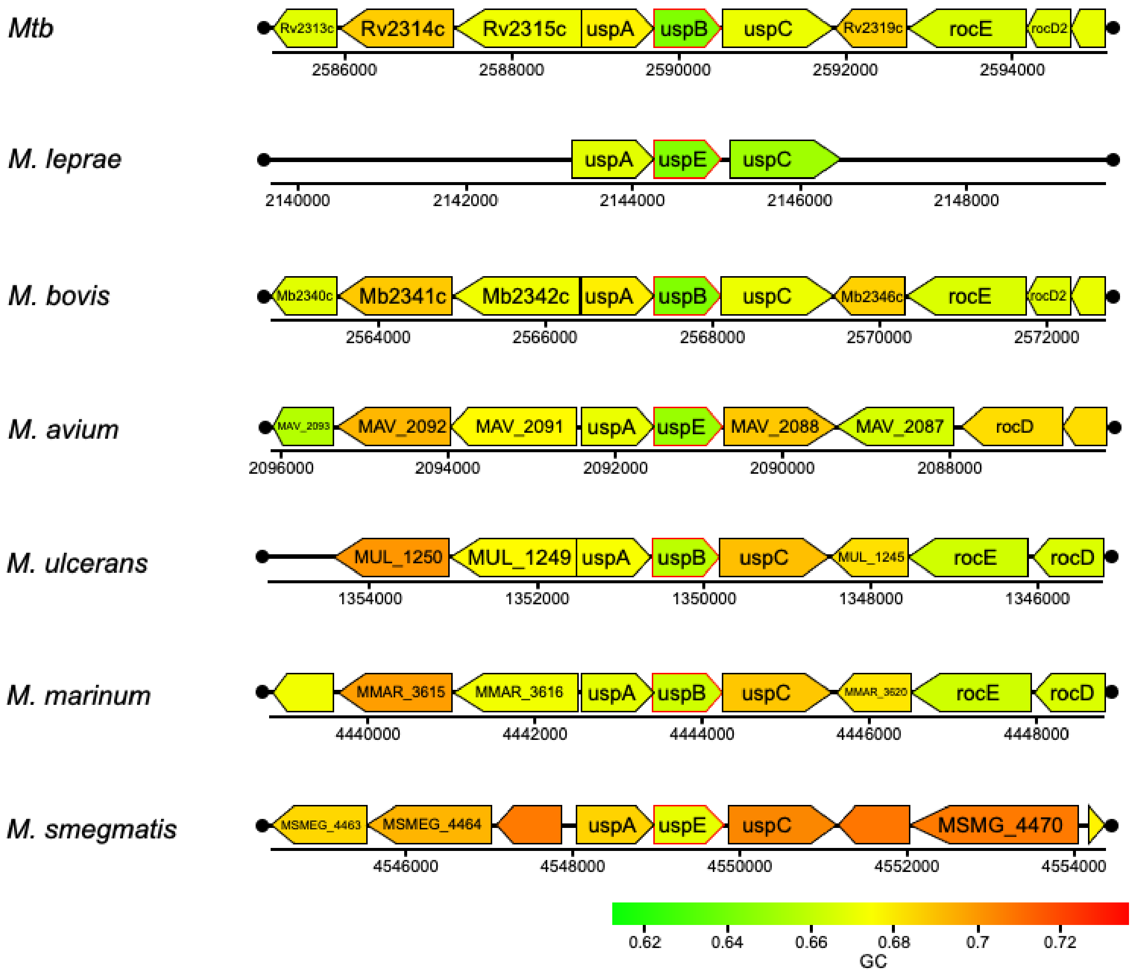

SI Fig. S2. **Transmembrane topology prediction of A) UspA B) UspB and C) UspC**, using the Hidden Markov model (TMHMM Server v2.0; Center for Biological Sequence Analysis, Technical University of Denmark (http://www.cbs.dtu.dk/services/TMHMM/).

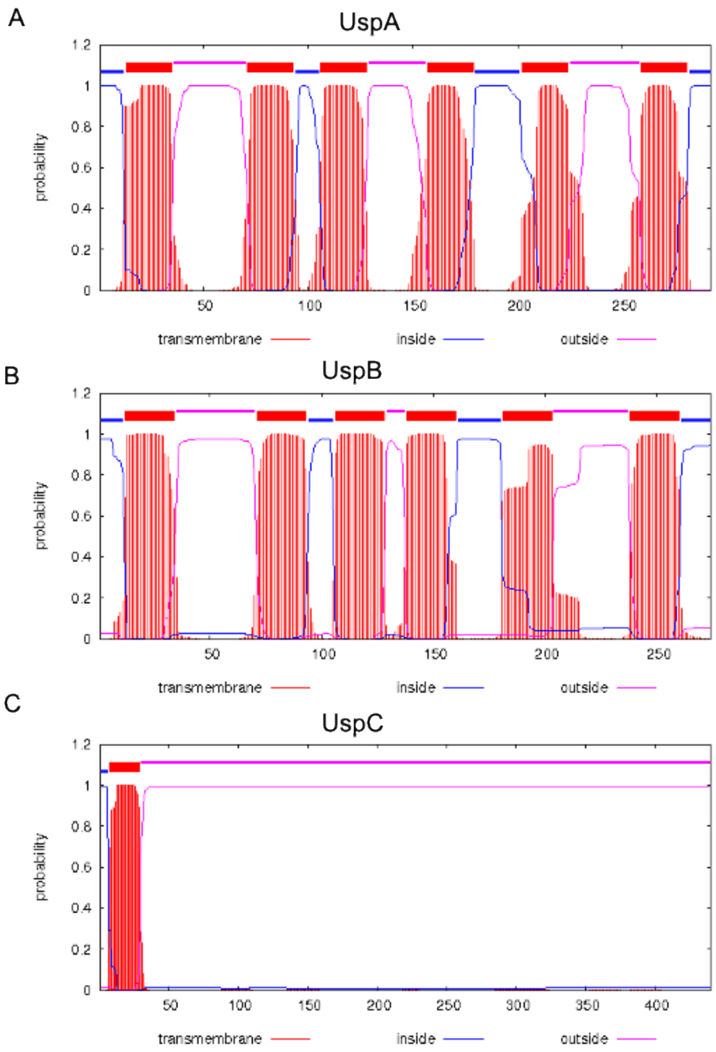

SI Fig. S3. Construction and confirmation of the *M. smegmatis* Δ*uspC* and Δ*uspAEC* mutants. A) Organisation of the *uspAEC* gene locus in *M. smegmatis* WT and the Δ*uspC* and Δ*uspAEC* mutants. γδres: res-sites of the δ-resolvase; hygR: hygromycin resistance gene. B) Whole genome sequencing analysis of Δ*uspC*. C) Whole genome sequencing analysis of Δ*uspAEC*

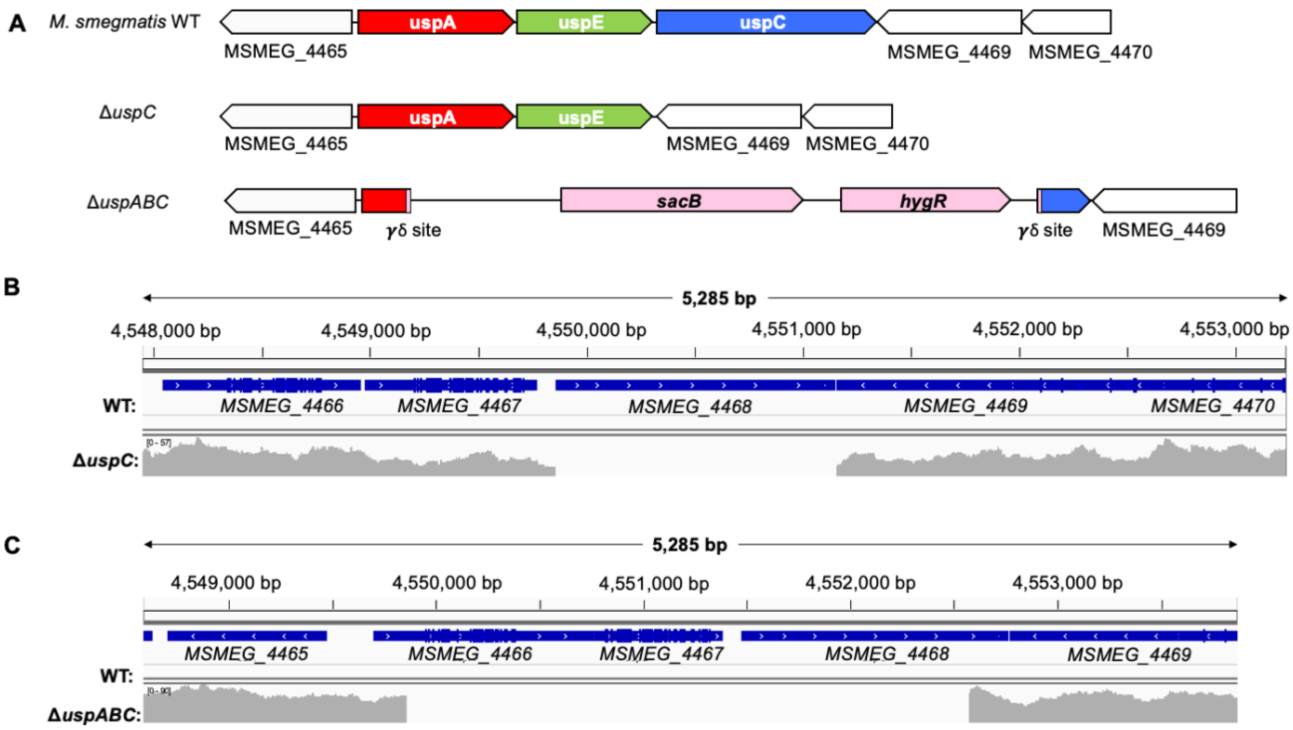

SI Fig. S4 Growth of *M. smegmatis*, *Mycobacterium smegmatis ΔuspC* and *M. smegmatis ΔuspAEC* mutants and complemented strains. A and B) Growth in Tryptic Soy Broth; C and D) Growth in Sauton's media. OD_{600} is the optical density at 600 nm. Data is representative of three biological replicates and the shaded area represents standard error of the mean.

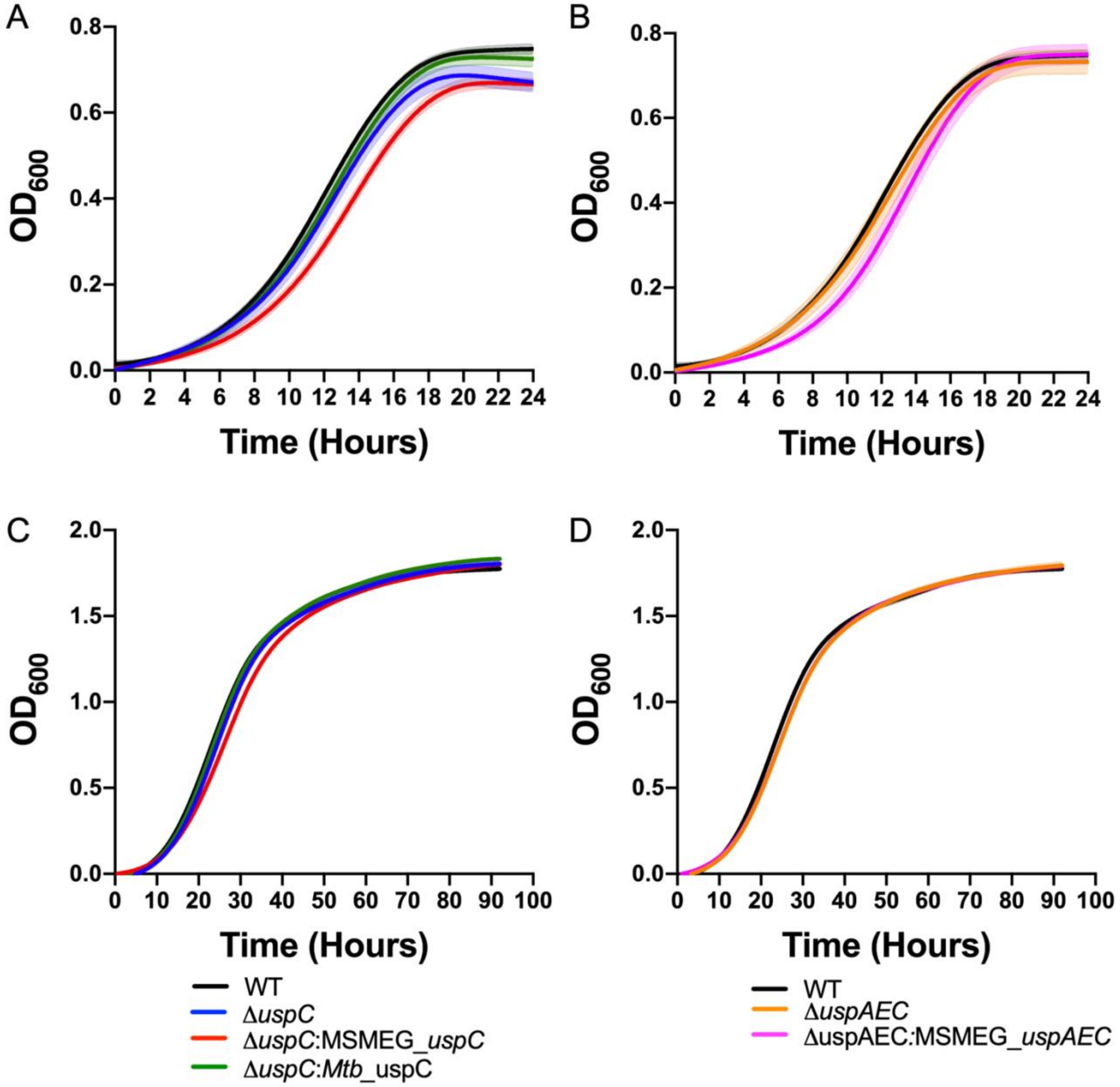

SI Fig. S5 Colony morphology and acid-fast staining of *M. smegmatis* WT, *M. smegmatis ΔuspC* and *Mycobacterium smegmatis ΔuspAEC* mutants and complemented strains. A). Ten-fold serial dilutions starting at 10^4 CFU of the *M. smegmatis* strains were spotted on LB agar and incubated at 37°C for five days. B). Acid-fast stained *M. smegmatis* strains.

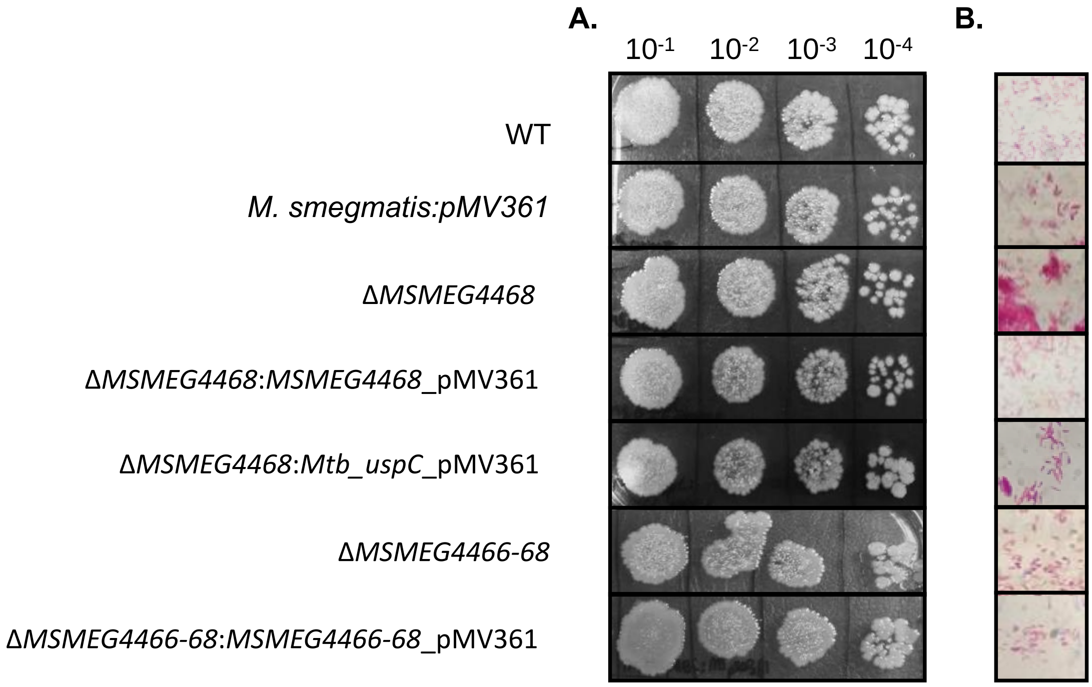

Fig S6. Plate map indicating the individual carbon sources for Biolog Phenotype MicorArray™ PM01 and PM02A (https://www.biolog.com/wp-content/uploads/2020/04/00A-042-Rev-C-Phenotype-MicroArrays-1-10-Plate-Maps.pdf)

PM1 MicroPlate™ Carbon Sources

A1<br>Negative Control	A2<br>L-Arabinose	A3<br>N-Acetyl-D-Glucosamine	A4<br>D-Saccharic Acid	A5<br>Succinic Acid	A6<br>D-Galactose	A7<br>L-Aspartic Acid	A8<br>L-Proline	A9<br>D-Alanine	A10<br>D-Trehalose	A11<br>D-Mannose	A12<br>Dulcitol
B1<br>D-Serine	B2<br>D-Sorbitol	B3<br>Glycerol	B4<br>L-Fucose	B5<br>D-Glucuronic Acid	B6<br>D-Gluconic Acid	B7<br>D,L-α-Glycerol-Phosphate	B8<br>D-Xylose	B9<br>L-Lactic Acid	B10<br>Formic Acid	B11<br>D-Mannitol	B12<br>L-Glutamic Acid
C1<br>D-Glucose-6-Phosphate	C2<br>D-Galactonic Acid-γ-Lactone	C3<br>D,L-Malic Acid	C4<br>D-Ribose	C5<br>Tween 20	C6<br>L-Rhamnose	C7<br>D-Fructose	C8<br>Acetic Acid	C9<br>α-D-Glucose	C10<br>Maltose	C11<br>D-Melibiose	C12<br>Thymidine
D-1<br>L-Asparagine	D2<br>D-Aspartic Acid	D3<br>D-Glucosaminic Acid	D4<br>1,2-Propanediol	D5<br>Tween 40	D6<br>α-Keto-Glutaric Acid	D7<br>α-Keto-Butyric Acid	D8<br>α-Methyl-D-Galactoside	D9<br>α-D-Lactose	D10<br>Lactulose	D11<br>Sucrose	D12<br>Uridine
E1<br>L-Glutamine	E2<br>m-Tartaric Acid	E3<br>D-Glucose-1-Phosphate	E4<br>D-Fructose-6-Phosphate	E5<br>Tween 80	E6<br>α-Hydroxy Glutaric Acid-γ-Lactone	E7<br>α-Hydroxy Butyric Acid	E8<br>β-Methyl-D-Glucoside	E9<br>Adonitol	E10<br>Maltotriose	E11<br>2-Deoxy Adenosine	E12<br>Adenosine
F1<br>Glycyl-L-Aspartic Acid	F2<br>Citric Acid	F3<br>m-Inositol	F4<br>D-Threonine	F5<br>Fumaric Acid	F6<br>Bromo Succinic Acid	F7<br>Propionic Acid	F8<br>Mucic Acid	F9<br>Glycolic Acid	F10<br>Glyoxylic Acid	F11<br>D-Cellobiose	F12<br>Inosine
G1<br>Glycyl-L-Glutamic Acid	G2<br>Tricarballylic Acid	G3<br>L-Serine	G4<br>L-Threonine	G5<br>L-Alanine	G6<br>L-Alanyl-Glycine	G7<br>Acetoacetic Acid	G8<br>N-Acetyl-β-D-Mannosamine	G9<br>Mono Methyl Succinate	G10<br>Methyl Pyruvate	G11<br>D-Malic Acid	G12<br>L-Malic Acid
H1<br>Glycyl-L-Proline	H2<br>p-Hydroxy Phenyl Acetic Acid	H3<br>m-Hydroxy Phenyl Acetic Acid	H4<br>Tyramine	H5<br>D-Psicose	H6<br>L-Lyxose	H7<br>Glucuronamide	H8<br>Pyruvic Acid	H9<br>L-Galactonic Acid-γ-Lactone	H10<br>D-Galacturonic Acid	H11<br>Phenylethyl-amine	H12<br>2-Aminoethanol

PM2A MicroPlate™ Carbon Sources

A1<br>Negative Control	A2<br>Chondroitin Sulfate C	A3<br>α-Cyclodextrin	A4<br>β-Cyclodextrin	A5<br>γ-Cyclodextrin	A6<br>Dextrin	A7<br>Gelatin	A8<br>Glycogen	A9<br>Inulin	A10<br>Laminarin	A11<br>Mannan	A12<br>Pectin
B1<br>N-Acetyl-D-Galactosamine	B2<br>N-Acetyl-Neuraminic Acid	B3<br>β-D-Allose	B4<br>Amygdalin	B5<br>D-Arabinose	B6<br>D-Arabitol	B7<br>L-Arabitol	B8<br>Arbutin	B9<br>2-Deoxy-D-Ribose	B10<br>i-Erythritol	B11<br>D-Fucose	B12<br>3-0-β-D-Galacto-pyranosyl-D-Arabinose
C1<br>Gentiobiose	C2<br>L-Glucose	C3<br>Lactitol	C4<br>D-Melezitose	C5<br>Maltitol	C6<br>a-Methyl-D-Glucoside	C7<br>β-Methyl-D-Galactoside	C8<br>3-Methyl Glucose	C9<br>β-Methyl-D-Glucuronic Acid	C10<br>α-Methyl-D-Mannoside	C11<br>β-Methyl-D-Xyloside	C12<br>Palatinose
D1<br>D-Raffinose	D2<br>Salicin	D3<br>Sedoheptulosan	D4<br>L-Sorbose	D5<br>Stachyose	D6<br>D-Tagatose	D7<br>Turanose	D8<br>Xylitol	D9<br>N-Acetyl-D-Glucosaminitol	D10<br>γ-Amino Butyric Acid	D11<br>δ-Amino Valeric Acid	D12<br>Butyric Acid
E1<br>Capric Acid	E2<br>Caproic Acid	E3<br>Citraconic Acid	E4<br>Citramalic Acid	E5<br>D-Glucosamine	E6<br>2-Hydroxy Benzoic Acid	E7<br>4-Hydroxy Benzoic Acid	E8<br>β-Hydroxy Butyric Acid	E9<br>γ-Hydroxy Butyric Acid	E10<br>a-Keto-Valeric Acid	E11<br>Itaconic Acid	E12<br>5-Keto-D-Gluconic Acid
F1<br>D-Lactic Acid Methyl Ester	F2<br>Malonic Acid	F3<br>Melibionic Acid	F4<br>Oxalic Acid	F5<br>Oxalomalic Acid	F6<br>Quinic Acid	F7<br>D-Ribono-1,4-Lactone	F8<br>Sebacic Acid	F9<br>Sorbic Acid	F10<br>Succinamic Acid	F11<br>D-Tartaric Acid	F12<br>L-Tartaric Acid
G1<br>Acetamide	G2<br>L-Alaninamide	G3<br>N-Acetyl-L-Glutamic Acid	G4<br>L-Arginine	G5<br>Glycine	G6<br>L-Histidine	G7<br>L-Homoserine	G8<br>Hydroxy-L-Proline	G9<br>L-Isoleucine	G10<br>L-Leucine	G11<br>L-Lysine	G12<br>L-Methionine
H1<br>L-Ornithine	H2<br>L-Phenylalanine	H3<br>L-Pyroglutamic Acid	H4<br>L-Valine	H5<br>D,L-Carnitine	H6<br>Sec-Butylamine	H7<br>D.L-Octopamine	H8<br>Putrescine	H9<br>Dihydroxy Acetone	H10<br>2,3-Butanediol	H11<br>2,3-Butanone	H12<br>3-Hydroxy 2-Butanone

Table S1. Unique carbon sources that result in substantial dye reduction (signal ≥ 0.2) for *M. smegmatis* Δ*uspC* and Δ*uspAEC* mutants

Plate/well position	Δ*uspC*	Plate/well position	Δ*uspAEC*
PM01 G7	Acetoacetic acid	PM01 B5	D-Glucuronic acid
PM01 G10	Methyl pyruvate	PM01 B7	D,L-α-Glycerol-phosphate
PM01 H10	D-Galacturonic acid	PM01 F8	Mucic acid
PM01 G12	L-Malic Acid	PM01 F9	Glycolic acid
PM02A F1	D-Lactic acid methyl ester	PM01 D10	Lactulose
PM02A D10	y-Amino butyric acid	PM01 F10	Glyoxylic acid
PM02A D12	Butyric acid	PM01 D12	Uridine
PM02A E12	5-Keto-D-gluconic acid	PM01 E12	Adenosine
		PM02A D1	D-Raffinose
		PM02A G1	Acetamide
		PM02A A2	Chondroitin sulfate C
		PM02A B2	*N*-Acetyl-neuraminic acid
		PM02A C2	L-Glucose
		PM02A D2	Salicin
		PM02A B3	β-D-Allose
		PM02A C3	Lactitol
		PM02A D3	Sedoheptulosan
		PM02A A4	β-Cyclodextrin
		PM02A B4	Amygdalin
		PM02A C4	D-Melezitose
		PM02A A5	y-Cyclodextrin
		PM02A C5	Maltitol
		PM02A A6	Dextrin
		PM02A C6	α-Methyl-D-glucoside
		PM02A E6	2-Hydroxy benzoic acid
		PM02A A7	Gelatin
		PM02A C7	β-Methyl-D-galactoside
		PM02A D7	Turanose
		PM02A E7	4-Hydroxy benzoic acid
		PM02A A8	Glycogen
		PM02A B8	Arbutin
		PM02A C8	3-Methyl glucose
		PM02A C9	β-Methyl-D-glucuronic acid
		PM02A G9	L-Isoleucine
		PM02A A11	Mannan
		PM02A G11	L-Lysine
		PM02A G12	L-Methionine
		PM02A H12	3-Hydroxy 2-butanone

Table S1. Primers used in this study. Restriction enzyme sites are shown in italics.

Name	Sequence
4466-F	CGTCCAGCACCTCTACTTCC
4467-R	CACCAGTTGTCCCAGCAG
4467-F	AGTGGACGCTGGTTATGG
4468-R	GGCGAAGTATCCGTTGGAG
MtbuspA-F	GTGGTGCTGTTCGTCATCC
MtbuspB-R	TAGACCCAGAACAACGCATC
MtbuspB-F	CAGTGGACGCTGGTGATG
MtbuspC-R	GCGAAGTAGGCGTTGGATAG
MysA _Fwd	CGAGGACGAGGAAGAAGAAG
MysA_Rev	TTGTAGCCCTTGGTGTAGTCG
SigA_Fwd	ACACTACGACCAGCACCATC
SigA_RRev	GTGAGTTCGGCGTCCTTG
Up_*MSMEG4468*_pJG_5	AAAA*TTAATTAA*CGATCGTCCAGCACCTCTAC
Up_*MSMEG4468*_pJG_3	AAAA*CCTAGG*GCCCGCCCCCTGCCGCCGGAG
Down_*MSMEG4468*_pJG_5	AAAA*CCTAGG*TCAGCCGCGTCGCCGGGGCAG
Down_*MSMEG4468*_pJG_3	AAAA*GGCGCGCC*ATGACCGTCGTGGTCATCTC
Up_*MSMEG4466-68*_pJG_5	AAAA*TTAATTAA*ATGCCGTTGGACTTGTAGGAG
Up_*MSMEG4466-68*_pJG_3	AAAA*CCTAGG*GCGCCGACGGGCCCATCACC
*MSMEG4466-68*_LL	TTTTTTTT*CAGAAACTG*GGTTCACCACCCAGTCCT
*MSMEG4466-68*_LR	TTTTTTTT*CAGTTCCTG*CCACCAGGGCCATGAACA
*MSMEG4466-68*_RL	TTTTTTTT*CAGAGACT*GGCGGGAGGTATTGGCGTG
*MSMEG4466-68*_RR	TTTTTTTT*CAGCTTCT*GTTGTCGTGGCCCGGTTGG
OL	CGGCCGATAATACGACTCA
HL	AGGATCCAGGACCTGCCAAT
OR	CTGACGCTCAGTCGAACGAA
HR	CTTCACCGATCCGGAGGAAC
MSMEG466-68KO_LHS_F	GTCTTGGGGACGTAGGACTG
MSMEG466-68KO_RHS_R	CATCCATCAACTGTGGGCG
blaTEM_101c_F	AAAA*AAGCTT*TACCAGAAACGCTGGTGAAAG
blaTEM_101c_R	AAAA*AAGCTT*TTACCAATGCTTAATCAGTGAG
*MSMEG4468*_pMV361_Fwd	AATT*GAATTC*ATGAGGCGCTCGACGCTGCT
*MSMEG4468*_pMV361_Rev	GATCGATC*AAGCTT*TCAACGTGATGCCGCCGCGT
*MSMEG4466-68*_pMV361_Fwd	AATT*GAATTC*GTGGCGCCGTGGCGGTTGCT
*MSMEG4468*_pMV361_Rev	GATCGATC*AAGCTT*TCAACGTGATGCCGCCGCGT
uspA_pmz102c_F	AAAA*GGATCC*AATGCGCGACGCGCCACGTCGG
uspA_pmz102c_R	AAAA*GAATTC*GGTGAGCTCATAGCTGATCC
uspB_pmz102c_F	AAAA*GGATCC*AATGAGCTCACCTAGTCGCGTC
uspB_pmz102c_R	AAAA*GAATTC*CTTGAGCCCCGAGACCACAATCG
uspC_pmz102c_F	AAAA*GGATCC*AGTGACCCGGCCCCGCCAGTCCACG
uspC_pmz102c_R	AAAA*GAATTC*GCGCTGTGTGGCAGCATTGG
uspA_pmz101_F	AAAA*GAATTC*TCATGCGCGACGCGCCACGTCGG
uspA_pmz101_R	AAAA*AAGCTT*CTAGGTGAGCTCATAGCTGATCC
uspB_pmz101_F	AAAA*GAATTC*TCATGAGCTCACCTAGTCGCGTCTCC
uspB_pmz101_R	AAAA*AAGCTT*TCACTTGAGCCCCGAGACCACAATCG
uspC_pmz101_F	AAAA*GAATTC*TCGTGACCCGGCCCCGCCAGTCC
uspC_pmz101_R	AAAA*AAGCTT*CTAGCGCTGTGTGGCAGCATTGG

Table S2. PM Additive solution. A) Composition and preparation of 120x stock solutions used for preparation of PM Additive solution. Stock solutions were prepared in sterile water, filter sterilised, and store at 4ºC. B) Composition and preparation of 100 mL of 12x PM Additive Solutions. Components were mixed from the 120x stock solutions, filter sterilised, and stored at 4ºC. C) Composition of the 1x PM Inoculating Fluid from stock solutions – used for addition to each well of the Biolog PM1 and PM2A plates, prepared under sterile conditions.

(A)

Stock solution	Ingredient	MW Weight	g/100ml	H_2O
$MgCl_2$-$CaCl_2$ stock	240 mM $MgCl_2.6H_2O$	203.3	4.88	100mL
	120 mM $CaCl_2.2H_2O$	147.0	1.76	
Tyloxapol	1.2 % Tyloxapol	-	1.2 (mL)	98.8mL

(B)

	12x PM stock
120 x stock: $MgCl_2$ and $CaCl_2$	10 ml
120 x stock: Tyloxapol	10 ml
Sterile H_2O	80 ml
Total	100 ml

(C)

PM Stock Solution	One plate (mL)
IF-0a GN/GP (1.2x)	10
Dye mix G (100x)	0.12
PM additive (12x)	1.0
M smegmatis cells (13.64x)	0.88
Total	12.0
